# Osteoid Metaplasia in Femoral Artery Plaques Is Associated With the Clinical Severity of Lower Extremity Artery Disease in Men

**DOI:** 10.3389/fcvm.2020.594192

**Published:** 2020-12-10

**Authors:** Mirjami Laivuori, Johanna Tolva, A. Inkeri Lokki, Nina Linder, Johan Lundin, Riitta Paakkanen, Anders Albäck, Maarit Venermo, Mikko I. Mäyränpää, Marja-Liisa Lokki, Juha Sinisalo

**Affiliations:** ^1^Department of Vascular Surgery, Helsinki University Hospital and University of Helsinki, Helsinki, Finland; ^2^Transplantation Laboratory, Department of Pathology, University of Helsinki, Helsinki, Finland; ^3^Department of Cardiology, Heart and Lung Center, Helsinki University Hospital and University of Helsinki, Helsinki, Finland; ^4^Translational Immunology Research Program, Research Programs Unit, University of Helsinki, Helsinki, Finland; ^5^Institute for Molecular Medicine Finland, HILIFE, University of Helsinki, Helsinki, Finland; ^6^Department of Global Public Health, Global Health/IHCAR, Karolinska Institutet, Stockholm, Sweden; ^7^Department of Pathology, HUSLAB, Meilahti Central Laboratory of Pathology, University of Helsinki, Helsinki, Finland

**Keywords:** atherosclerosis, lower extremity artery disease (LEAD), osteoid metaplasia, machine learning, digital pathology, histology, artificial intelligence

## Abstract

Lamellar metaplastic bone, osteoid metaplasia (OM), is found in atherosclerotic plaques, especially in the femoral arteries. In the carotid arteries, OM has been documented to be associated with plaque stability. This study investigated the clinical impact of OM load in femoral artery plaques of patients with lower extremity artery disease (LEAD) by using a deep learning-based image analysis algorithm. Plaques from 90 patients undergoing endarterectomy of the common femoral artery were collected and analyzed. After decalcification and fixation, 4-μm-thick longitudinal sections were stained with hematoxylin and eosin, digitized, and uploaded as whole-slide images on a cloud-based platform. A deep learning-based image analysis algorithm was trained to analyze the area percentage of OM in whole-slide images. Clinical data were extracted from electronic patient records, and the association with OM was analyzed. Fifty-one (56.7%) sections had OM. Females with diabetes had a higher area percentage of OM than females without diabetes. In male patients, the area percentage of OM inversely correlated with toe pressure and was significantly associated with severe symptoms of LEAD including rest pain, ulcer, or gangrene. According to our results, OM is a typical feature of femoral artery plaques and can be quantified using a deep learning-based image analysis method. The association of OM load with clinical features of LEAD appears to differ between male and female patients, highlighting the need for a gender-specific approach in the study of the mechanisms of atherosclerotic disease. In addition, the role of plaque characteristics in the treatment of atherosclerotic lesions warrants further consideration in the future.

## Introduction

Atherosclerosis is a chronic multifactorial disease characterized by progressing calcification of the vasculature (1). Previous research of atherosclerosis has been dominated by studies on coronary artery disease (CAD) with less attention given to lower extremity artery disease (LEAD) (2) caused by atherosclerosis of the lower limb arteries. Cardiovascular risk factors including older age, diabetes, dyslipidemia, cigarette smoking, and hypertension explain ~70% of the incidence of LEAD (3). The prevalence of LEAD increased by nearly 25% between 2000 and 2010 (4). Thus, the globally increasing incidence of LEAD warrants further understanding of the mechanisms and etiology of the disease.

Recently, artificial intelligence (AI) algorithms and deep learning (DL) have enabled unprecedented progress in histological analysis. DL-based methods provide an accurate method of characterizing and quantifying tissue entities in digitized histopathological samples (5, 6). The technological advances enabling the quantitative study of tissue samples have grown rapidly, and there is a call for translational research bridging the way to the clinical setting and patient care (7).

The morphological features of atherosclerotic plaques have been associated with clinical features and disease progression in coronary artery and carotid artery disease. The influence of calcification on plaque vulnerability has been studied in the carotid arteries by using imaging, and pathological and computational modeling technologies. The studies suggest that severe calcification might stabilize the plaque, whereas the early phase of calcification, called microcalcification in the thin fibrous cap, could have the opposite effect (8–10). Macroscopic calcium deposition (macrocalcification) in the coronary arteries also seems to be a feature of more stable plaques in imaging studies (11, 12).

The formation of metaplastic lamellar bone, known as osteoid metaplasia (OM), has been associated with advanced calcification of vasculature (13). Previously, OM has been reported in 51–65% of studied femoral artery plaques (14, 15), in 13–20% of carotid artery plaques (16–18), and in 13–15.6% of cardiac valves (19, 20). In the coronary arteries, OM is regarded to be an uncommon feature (21). It is still not fully understood why the OM load varies between arterial beds. One possible explanation could be that the femoral artery plaques extracted during surgery represent a late stage of atherosclerotic disease as compared with carotid artery plaques (2). Although an indicator of advanced calcification, histologically detected OM has been described to have protective effects against cerebrovascular events, (16) and it is a more common feature in stable than unstable carotid artery plaques (16). In LEAD patients, the link to clinical disease and the role of OM in the disease progression leading to symptoms remains less studied and poorly understood. Furthermore, the pathological quantification of calcification and OM in the plaques has mostly been imprecise or based on subjective visual estimation (10).

The aim of this study was to quantify the OM present in the digitized hematoxylin and eosin (H&E)-stained femoral endarterectomy specimens by building a DL algorithm. The results of the analysis were then used to study the association between the amount of OM in the plaque and clinical disease stage of LEAD as well as other clinical characteristics contributing to the prognosis of atherosclerotic disease.

## Materials and Methods

### Patient Samples

**Figure 1:**
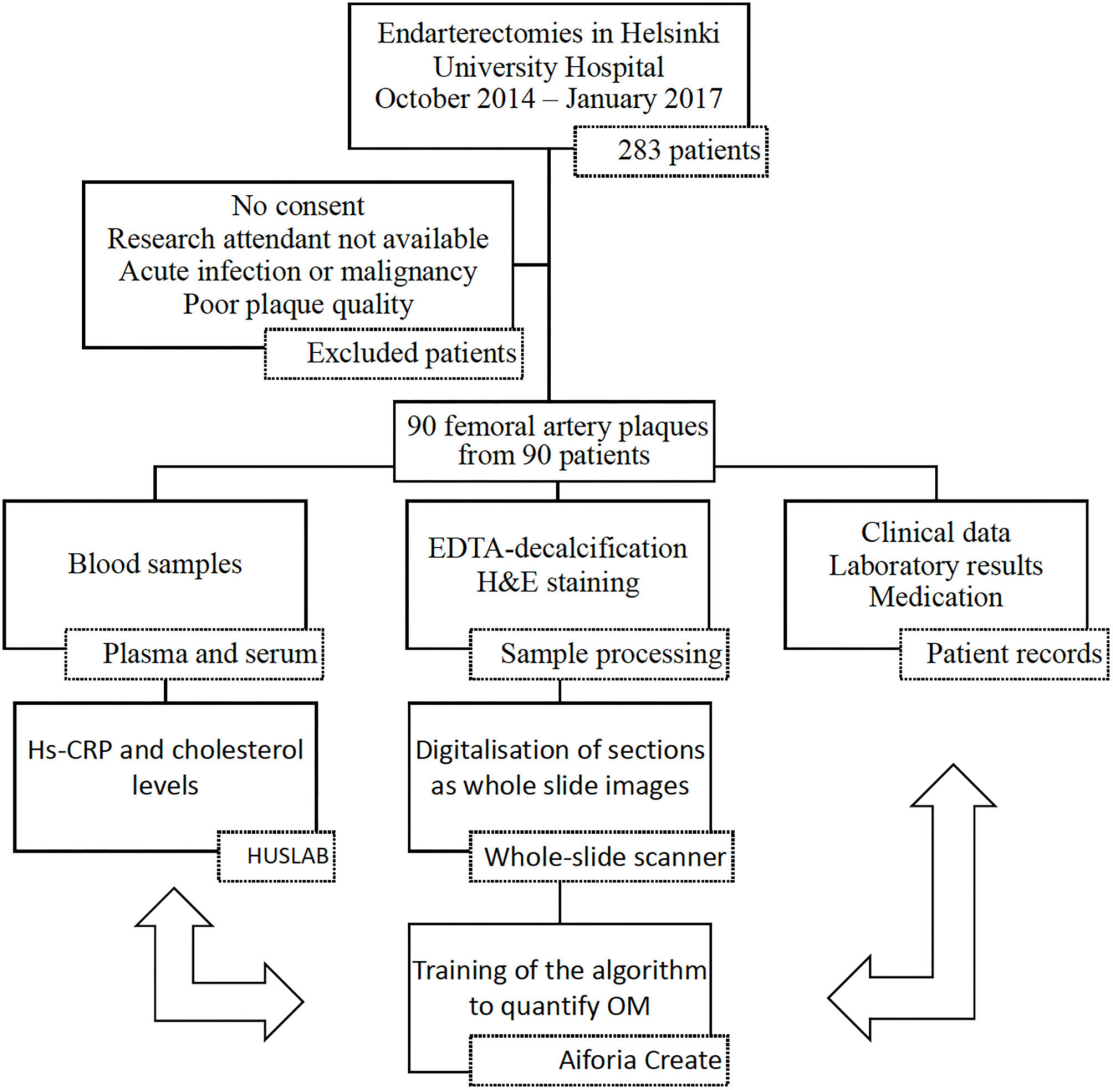
Workflow of the study. Data of the patients were collected in three ways: laboratory analysis from blood samples, study of the extracted plaque, and data collection from the electronic patient records. For the final analysis all the data were combined. hs-CRP, high-sensitivity C-reactive protein; HUSLAB, Helsinki University Hospital Laboratory; H&E, hematoxylin and eosin; DL, deep learning; OM, osteoid metaplasia. Plaques were collected from 97 patients. After the plaques were processed, samples from 90 patients were included in the final analysis.

The pipeline of patient recruitment, sample treatment, and analysis is presented in Figure 1. There were 283 endarterectomies of the left or right common femoral artery and/or its bifurcation performed at the Helsinki University Hospital between October 2014 and January 2017. Of them, the femoral artery plaques from 97 patients were collected. Written informed consent was required from patients. A prerequisite for including a patient in this study was that the research attendant was available during the surgery to collect the plaque. Patients with acute infection and active malignancy were excluded from the study. Clinical data were collected from the electronic patient records.

The Ethics Committee of the Hospital District of Helsinki and Uusimaa approved the protocol for this study (Dnro 78/13/03/00/2014), and the study conformed to the principles outlined in the Declaration of Helsinki.

The surgeon performed the endarterectomy according to standard procedure by removing the plaque from the common femoral artery and arterial bifurcation. The research attendant photographed the samples and cut the endarterectomy specimens immediately into smaller longitudinal samples for processing and set the middle section into 10% formaldehyde for fixing. Plaque samples were decalcified for up to 6 months using EDTA, a standard method for decalcifying mineralized samples that does not alter the morphology of the tissue (22). Decalcification was continued until processing of the sample was feasible, which was assessed by probing the specimens with a thin needle. The samples were embedded in paraffin, and the paraffin blocks were sectioned into 4-μm-thick longitudinal sections and stained with standard H&E protocol. H&E-stained sections of sufficient quality were available from 90 patients. The entire H&E slides were then digitized using a whole-slide scanner (3D HISTECH Pannoramic 250 Flash III, 3DHistec, Budapest, Hungary) with 20× objective and a pixel size of 0.23 μm. Thereafter, the whole-slide images (WSIs) were uploaded to a cloud-based image management and machine learning platform (Aiforia Create, Aiforia Technologies Oy, Helsinki, Finland) for image analysis algorithm development, training, and analysis.

### Deep Learning Algorithm

**Figure 2:**
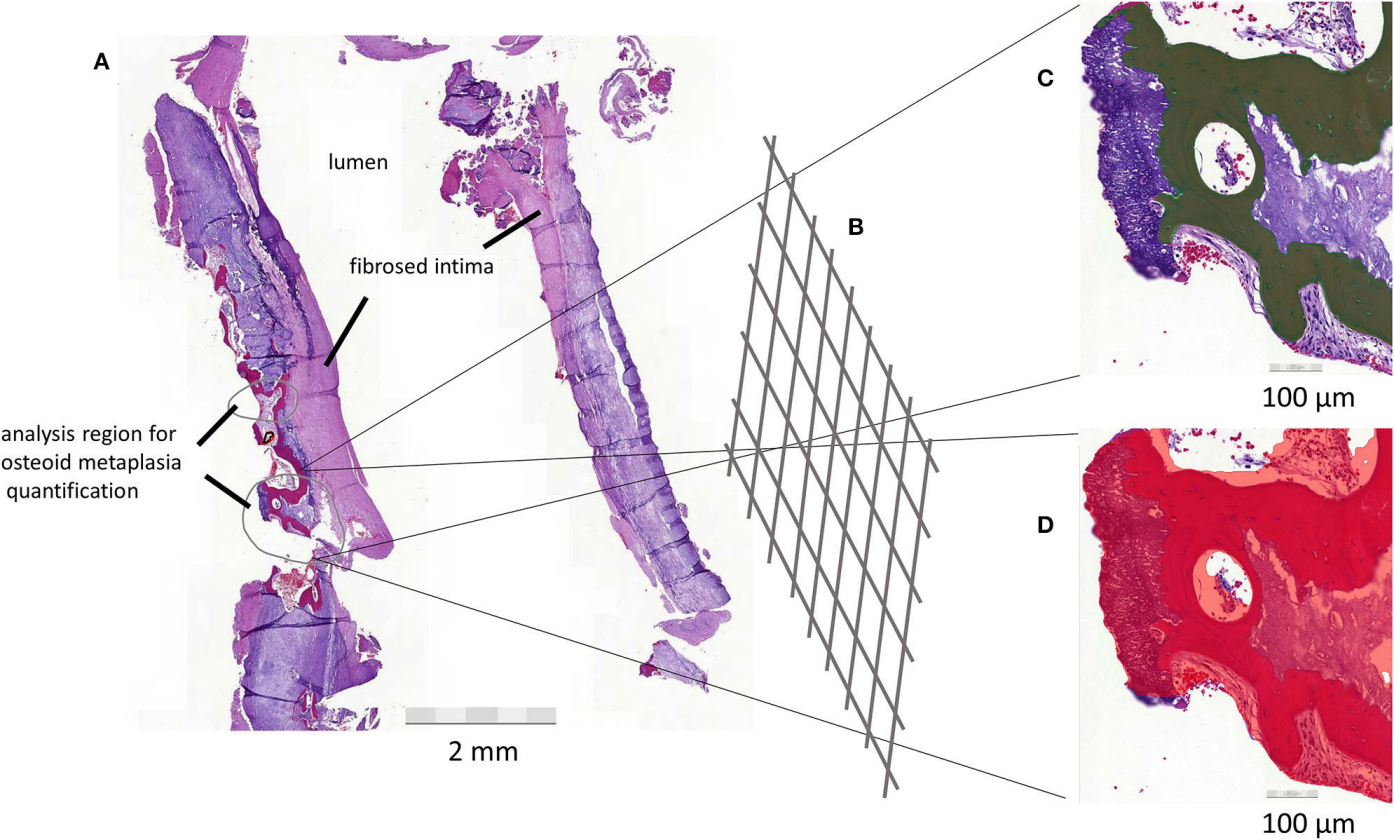
**(A)** Femoral artery plaque is sectioned and opened to expose the lumen of the artery. Analysis regions for algorithm building are circled in gray. **(B)** Deep neural network learning. **(C)** Osteoid metaplasia (OM), shown in dark color, is quantified with confidence level and color representation to allow for evaluation of the identified structures. **(D)** The final algorithm also evaluates the amount of tissue. This allows the proportional quantification of the target structure (OM) in overall tissue. The process is visualized in the figure with a plaque from one patient.

To quantify the area percentage of lamellar bone-like structures in a WSI, a DL-based image analysis algorithm was trained (Figure 2). The analysis algorithm was first trained to recognize tissue in the H&E slide. Next, a second layer was added to the algorithm to identify lamellar bone-like structures within the recognized tissue regions. Finally, the analysis was expanded to the whole plaque section to be able to calculate the area percentage of bone-like structure within the section.

### Training of the Deep Learning Algorithm

Forty-six WSIs were used for training the algorithm for the tissue layer. Training consists of tissue training and negative training to identify debris and background. Tissue detection was trained with a total of 378 annotations across 38 WSIs. Seventy-six WSIs were used for training of the OM layer. OM detection was trained with positive annotations for detecting OM and negative annotations for discriminating other tissue structures from OM. OM detection was trained with a total of 405 annotations across 44 WSIs. The following parameter settings were used in training the algorithm: for the tissue layer, region context size of 50 μm was used with complexity level “complex” and default image augmentation settings. For the OM layer, region context size was 70 μm with complexity level “extra complex” and improved image augmentation settings of −40 to +40 luminance and −40 to +40 contrast, with maximum white balance change 10 and noise level 2. The sensitivity of the final algorithm was 99.9% for tissue detection and 96.48% for OM, precision 99.8% and 96.5%, and F-score 0.999 and 0.961, respectively.

The algorithm was applied to the WSIs of atherosclerotic plaque section from 90 patients. The algorithm determined the size and class confidence percentage for each identified OM area. A weighted mean of area and class confidence percentage of each OM region was calculated for every slide. Finally, we performed a post-analysis quality control assessment. Two researchers (ML and JT) went independently through all the slides and visually confirmed the presence of OM on the slides, which the algorithm had counted as having OM. In case of conflicting decisions, a third researcher (IL) was consulted. The majority of the very small identified areas (length < 100 μm) were identified as false-positive annotations and not actual OM. The slides that were included in the final analysis as having OM were those that had a class confidence weighted mean of at least 80%.

There were seven slides that the algorithm had identified as having OM, but all the areas in the slide were in fact thrombus by visual confirmation. These slides were not considered to have OM in the final analysis. In addition, there were five slides with weighted mean 78.1–79.1% that had OM based on visual confirmation despite the weighted mean under 80%. These slides were considered to have OM in the final analysis.

### Clinical Data

The relevant clinical data and medication including use of angiotensin-converting enzyme (ACE) inhibitors, acetylsalicylic acid, clopidogrel, angiotensin II receptor (AT) blockers, statins, and warfarin were extracted from the electronic patient records. Clinical symptoms were categorized according to the Fontaine classification of clinical presentation of LEAD (23). Due to the small sample size, we formed three symptom categories with increasing disease severity: claudication, rest pain, and ulcer or gangrene based on the Fontaine classification.

The ankle brachial index (ABI) was calculated by dividing the highest ankle pressure by the highest brachial systolic pressure. Toe pressure (TP) was the systolic blood pressure measured from the great toe. The ABI and TP measurements were carried out according to current guidelines by experienced nurses (24).

### Blood Samples and Comorbidities

Serum, EDTA, and citrate plasma samples were collected from patients and stored at −80°C until analysis. All the biochemical parameter analyses were conducted at the Helsinki University Hospital Laboratory (HUSLAB). Total leukocyte count (TLC), thrombocytes, creatinine, and hemoglobin (Hb) were measured on the day of surgery or 1–21 days before the surgery, whereas total cholesterol (TC), high-density lipoprotein-cholesterol (HDL-C), low-density lipoprotein-cholesterol (LDL-C), triglycerides (TG), and high-sensitivity C-reactive protein (hs-CRP) were analyzed from the blood sample drawn on the day of surgery. Glomerular filtration rate (GFR) was calculated according to the Chronic Kidney Disease Epidemiology Collaboration (CKD-EPI) (25) formula for male and female patients separately adjusted by the creatinine level.

Diabetes was defined by ICD-10 diagnosis code for diabetes (E10–E14 codes) in electronic patient records or by use of diabetes medication. Hypertension was defined as an ICD-10 diagnosis code for hypertension or use of antihypertensive drugs for the indication of hypertension. Dyslipidemia was defined as an ICD-10 diagnosis code for dyslipidemia, random plasma LDL-C level of >3 mmol/L or HDL-C level lower than 1 mmol/L in men and lower than 1.2 mmol/L in women. Cerebrovascular disease was defined as a history of stroke or transient ischemic attack. CAD was defined as a history of acute coronary syndrome or diagnostic findings in coronary angiography or exercise electrocardiography.

### Statistical Analyses

The statistical analysis was performed using SPSS 23 (SPSS Inc., Chicago, IL, USA). Associations between variables were determined with chi-square test, Fischer's exact, Mann–Whitney *U*-test, Kruskal–Wallis test, one-way ANOVA, and Spearman's rank-order correlation test, when appropriate. Continuous variables were expressed as mean ± standard deviation (SD) or median with interquartile range where appropriate and categorical variables as count (percentage).

Firstly, associations between presence of OM as a binary categorical variable and clinical parameters were computed. Secondly, correlations between OM and clinical data were determined using OM area percentage as a continuous variable. All the analyses were performed for all of the cases and separately for male and female patients.

Thirdly, the continuous OM variable was categorized separately for male and female patients in order to create three groups according to the area percentage of OM in the slide. The first group included patients without OM (lowest group). The patients with OM were divided into two groups based on the median of the area percentages of OM in the slides (middle and highest groups). A Mantel–Haenszel linear-by-linear association test for trend for categorical variables and Jonckheere–Terpstra test for trend for continuous variables were used to examine whether the groups differed statistically regarding the clinical parameters.

Finally, a regression analysis was performed to evaluate the relationship between the area percentage of OM and clinical symptoms (claudication vs. rest pain or ulcer) as a dependent variable and OM (the lowest, middle, and highest groups) as an explanatory variable. The equation was adjusted for TP and thrombocytes, which were independently associated with symptoms in male patients. A multivariable adjusted logistic regression model, adjusted for TP, thrombocytes, and cardiovascular risk factors (age, diabetes, dyslipidemia, cigarette smoking, and hypertension), was also performed. Additionally, another multivariable logistic regression model without dyslipidemia was analyzed. We considered that dyslipidemia might skew the results because the usage of statin therapy or the duration of statin therapy may affect the effect of dyslipidemia, which was not observed in the study. Symptoms were analyzed using only two categories, claudication and rest pain/ulcer, because of the small number of patients in the subgroup analyses.

Statistical significance of results was set at *p* < 0.05.

## Results

The H&E-stained slides showed variation in plaque composition and structural dysmorphia (Supplementary Figures I, II). The OM structures were detectable by eye due to their distinct lamellated appearance (Supplementary Figure III). OM was observed in morphologically varying plaques, and 56.7% of the plaques had OM.

### Baseline Characteristics

The patients were on average 69.6 years old with 37.8% being female. Baseline characteristics are presented in detail in Table 1 with separate columns for the whole cohort, patients with and patients without OM. No significant differences were evident between these groups. Body mass index (BMI), diabetes, smoking status, use of clopidogrel, hemoglobin (Hb), GFR, and cholesterol levels differed significantly between female and male patients (Table 2).

**Table 1:** Baseline characteristics and their associations with presence of OM.

**Characteristic**	**Total**	**OM–**	**OM+**	***p***
*N*	90	39 (43.3)	51 (56.7)	
Female	34 (37.8)	17 (43.6)	17 (33.3)	0.320
Age (years)	69.34 [9.41]	68.62 [8.96]	69.42 [10.15]	0.471
Age range (years)	50.12–84.43	50.12–83.27	54.46–79.23	
BMI (kg/m^2^) (*n* = 74)	26.10 [7.00]	26.25 [7.00]	25.95 [7.00]	0.289
Previous invasive treatments for LEAD	40 (44.4)	19 (48.7)	21 (41.2)	0.476
Severity of LEAD symptoms				
Claudication	57 (63.3)	28 (71.8)	29 (56.9)	0.145
Rest pain	18 (20.0)	7 (17.9)	11 (21.6)	0.671
Ischemic ulcer or gangrene	15 (16.7)	4 (10.3)	11 (21.6)	0.154
Ankle brachial index (*n* = 76)	0.42 [0.21]	0.40 [0.18]	0.45 [0.24]	0.847
Toe pressure (mmHg) (*n* = 76)	41.00 [26.00]	40.00 [29.00]	45.00 [20.00]	0.588
**Risk factors**				
Smoking status (*n* = 88)				
Never	4 (4.5)	3 (7.9)	1 (2.0)	0.311
Current smoker	39 (44.3)	16 (42.1)	23 (46.0)	0.716
Ex-smoker	45 (51.1)	19 (50.0)	26 (52.0)	0.853
Diabetic	31 (34.4)	10 (25.6)	21 (41.2)	0.124
Hypertensive	76 (84.4)	31 (79.5)	45 (88.2)	0.256
Dyslipidemia (*n* = 89)	81 (91.0)	34 (87.2)	47 (94.0)	0.291
Coronary artery disease	21 (34.4)	12 (30.8)	19 (37.3)	0.521
Cerebrovascular disease	8 (8.9)	3 (7.7)	5 (9.8)	0.727
**Medications**				
ACE inhibitors/ATR blockers	67 (74.4)	26 (66.7)	41 (80.4)	0.139
Aspirin	65 (72.2)	29 (74.4)	36 (70.6)	0.692
Clopidogrel	10 (11.1)	3 (7.7)	7 (13.7)	0.505
Statins	64 (71.1)	27 (69.2)	37 (72.5)	0.731
Warfarin	5 (5.6)	1 (2.6)	4 (7.8)	0.384
**Laboratory results**				
Hemoglobin (g/L) (*n* = 88)	138.00 [22.30]	138.00 [20.00]	138.00 [25.00]	0.997
Total leukocyte count (10E9/L) (*n* = 88)	7.90 [2.70]	7.70 [2.80]	8.00 [2.50]	0.896
Thrombocytes (10E9/L) (*n* = 88)	261.00 [89.80]	261.00 [95.50]	261.00 [91.00]	0.534
Total cholesterol (mmol/L) (*n* = 88)	4.10 [1.10]	3.90 [1.33]	4.10 [1.05]	0.631
Low-density lipoprotein-cholesterol (mmol/L) (*n* = 87)	2.10 [1.00]	2.00 [0.90]	2.10 [1.20]	0.826
High-density lipoprotein-cholesterol (mmol/L) (*n* = 88)	1.21 [0.50]	1.12 [0.50]	1.25 [0.50]	0.649
Triglycerides (mmol/L) (*n* = 88)	1.28 [0.71]	1.18 [0.75]	1.28 [0.54]	0.830
High-sensitivity C-reactive protein (mg/L) (*n* = 88)	1.67 [2.58]	1.54 [1.85]	1.88 [3.40]	0.584
Glomerular filtration rate (μmol/L) (*n* = 88)	81.34 [19.86]	80.50 [22.86]	82.45 [19.92]	0.597

*Note. Data are presented as median [IQR] or number of study subjects in the column (%). p-value refers to the statistical significance of the difference in each characteristic between the patients who had OM and those who did not. Information of each clinical parameter was available for all the patients unless otherwise stated*.

*OM, osteoid metaplasia; OM–, patients without OM; OM+, patients with OM; BMI, body mass index; LEAD, lower extremity artery disease*.

**Table 2:** Differences in baseline characteristics between female and male patients.

**Characteristic**	**Female**	**Male**	***p***
No. of patients with OM	17 (50)	34 (60.7)	0.383
Amount of OM	0.200 ± 0.42	0.153 ± 0.28	0.955
Age (years)	70.96 [11.72]	68.58 [10.15]	0.068
Age range (years)	59.93–83.27	50.12–84.43	
BMI (kg/m^2^)	23.23 [7.00]	27.76 [7.00]	<0.0001
Previous invasive treatment for LEAD	16 (47.1)	25 (43.9)	0.767
Severity of LEAD symptoms			0.455^a^
Claudication	22 (64.7)	35 (61.4)	
Rest pain	8 (23.5)	10 (17.5)	
Ischemic ulcer or gangrene	4 (11.8)	12 (21.1)	
Ankle brachial index	0.40 [0.16]	0.45 [0.24]	0.396
Toe pressure (mmHg)	39.00 [24.00]	45.00 [22.00]	0.548
**Risk factors**			
Smoking status (*n* = 88)			0.002^a^
Never	4 (11.8)	0	
Current smoker	11 (32.4)	34 (59.6)	
Ex-smoker	18 (52.9)	22 (38.6)	
Diabetic	6 (17.6)	26 (45.6)	0.007
Dyslipidemia (n = 89)	29 (85.3)	53 (93.0)	0.131
Coronary artery disease	11 (32.4)	21 (36.8)	0.664
Cerebrovascular disease	4 (11.8)	4 (7.0)	0.466
**Medications**			
ACE inhibitors/ATR blockers	22 (64.7)	46 (80.7)	0.089
Aspirin	25 (73.5)	41 (71.9)	0.869
Clopidogrel	8 (23.5)	2 (3.5)	0.005
Statins	24 (70.6)	41 (71.9)	0.891
Warfarin	0	5 (8.8)	0.153
**Laboratory results**			
Hemoglobin (g/L) (*n* = 88)	130.00 [12.00]	142.00 [23.00]	0.008
Total leukocyte count (10E9/L) (*n* = 88)	7.70 [2.80]	7.90 [2.40]	0.221
Thrombocytes (10E9/L) (*n* = 88)	269.00 [90.50]	251.00 [82.00]	0.472
Total cholesterol (mmol/L) (*n* = 88)	4.35 [1.60]	3.85 [1.30]	0.002
Low-density lipoprotein-cholesterol (mmol/L) (*n* = 87)	2.40 [1.30]	1.95 [0.90]	0.030
High-density lipoprotein-cholesterol (mmol/L) (*n* = 88)	1.36 [0.30]	1.07 [0.40]	<0.0001
Triglycerides (mmol/L) (*n* = 88)	1.13 [0.39]	1.30 [1.19]	0.048
High-sensitivity C-reactive protein (mg/L) (*n* = 88)	1.39 [1.34]	1.82 [3.72]	0.276
Glomerular filtration rate (ml/min/1.73 m^2^) (*n* = 88)	76.67 [17.68]	87.36 [20.47]	0.019

*Note. Data are presented as median [IQR] or number of study subjects (%). Information of each clinical parameter was available for all the patients unless otherwise stated*.

*OM, osteoid metaplasia; BMI, body mass index; LEAD, lower extremity artery disease*.

a *Linear by linear association between 3 smoking/symptom groups*.

### Presence of Osteoid Metaplasia in the Slide

There was no statistical difference in the incidence of OM between female (*n* = 17, 50.0%) and male (*n* = 34, 60.7%) patients (*p* = 0.320). In female patients, the incidence of OM was associated with diabetes (*p* = 0.018) and higher TP (*p* = 0.043), and previous invasive treatment for LEAD in male patients (OR = 0.331; 95%CI = 0.109–1.008, *p* = 0.048). All diabetic women in the study had OM (Supplementary Table I).

### Quantification of Osteoid Metaplasia

**Figure 3:**
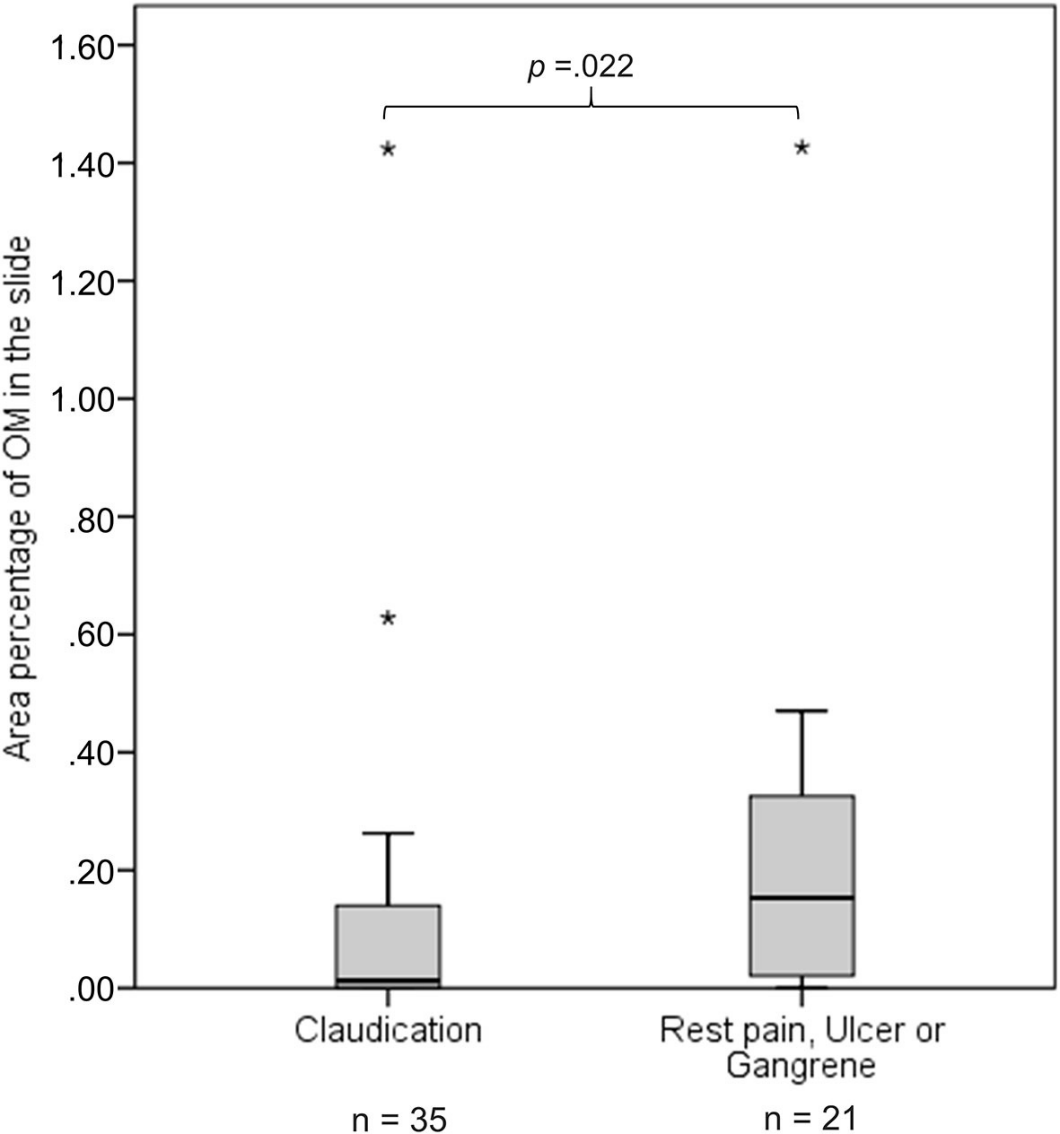
The figure presents as boxplots the area percentage of osteoid metaplasia (OM) in a slide for patients with claudication and patients with rest pain, ulcer, or gangrene. Area percentage of OM in the slide differs significantly between male patients (*n* = 56) with claudication and male patients with rest pain, ulcer, or gangrene. *p* is calculated using Mann–Whitney *U*-test. Outliers are marked with stars.

Table 3 shows the association between clinical characteristics and area percentage of OM in a slide for categorical variables and Table 4 for continuous variables. The area percentage of OM was not associated with clinical characteristics in the whole cohort analysis. When females and males were analyzed separately, the area percentage of OM associated significantly with diabetes in the female patients. The females with diabetes had more OM compared with the females without diabetes (mean ± SD, 0.547% ± 0.880 vs. 0.130% ± 0.193, respectively, *p* = 0.029). In male patients, the area percentage of OM correlated inversely with TP (correlation coefficient −0.296, *p* = 0.044) and was significantly associated with symptoms (mean ± SD, 0.113% ± 0.261 for patients with claudication vs. 0.220% ± 0.316 for patients with rest pain or ulcer, *p* = 0.022) (Figure 3).

**Table 3:** Association of the area percentage of OM to categorical baseline characteristics.

**Characteristic**	**All patients**			**Female**			**Male**		
	**Yes**	**No**	***p***	**Yes**	**No**	***p***	**Yes**	**No**	***p***
Previous invasive treatments for LEAD	0.192 (0.428)	0.158 (0.256)	0.670	0.301 (0.566)	0.116 (0.196)	0.136	0.119 (0.297)	0.183 (0.285)	0.057
Severity of LEAD symptoms			0.204^a^			0.977^a^			0.066^a^
Claudication	0.131 (0.241)			0.155 (0.203)			0.113 (0.261)		
Rest pain	0.148 (0.155)			0.118 (0.160)			0.158 (0.156)		
Ischemic ulcer or gangrene	0.377 (0.675)			0.609 (1.143)			0.275 (0.413)		
Claudication vs. rest pain/ulcer/gangrene^b^	0.131 (0.241)	0.252 (0.473)	0.101	0.155 (0.203)	0.281 (0.657)	0.894	0.113 (0.261)	0.220 (0.316)	0.022
**Risk factors**									
Smoking status (*n* = 88)			0.262^a^			0.350^a^			0.400^a^
Never	0.028 (0.057)			0.028 (0.057)			N/A		
Current smoker	0.256 (0.479)			0.278 (0.547)			0.236 (0.422)		
Ex-smoker	0.118 (0.165)			0.151 (0.185)			0.107 (0.160)		
Diabetes	0.199 (0.434)	0.161 (0.293)	0.363	0.547 (0.880)	0.130 (0.193)	0.029	0.109 (0.147)	0.188 (0.360)	0.745
Dyslipidemia (*n* = 89)	0.182 (0.357)	0.093 (0.169)	0.341	0.216 (0.452)	0.146 (0.201)	0.882	0.163 (0.296)	0.004 (0.007)	0.150
Coronary artery disease	0.123 (0.166)	0.202 (0.410)	0.951	0.090 (0.132)	0.264 (0.502)	0.371	0.141 (0.183)	0.162 (0.339)	0.412
Cerebrovascular disease	0.155 (0.177)	0.176 (0.356)	0.778	0.199 (0.250)	0.206 (0.438)	1.000	0.122 (0.134)	0.157 (0.299)	0.648
**Medications**									
ACE inhibitors/ATR blockers	0.179 (0.352)	0.160 (0.330)	0.286	0.246 (0.502)	0.135 (0.228)	0.530	0.146 (0.250)	0.188 (0.425)	0.433
Aspirin	0.174 (0.361)	0.173 (0.304)	0.765	0.235 (0.484)	0.128 (0.174)	0.838	0.137 (0.259)	0.200 (0.364)	0.538
Clopidogrel	0.190 (0.169)	0.172 (0.360)	0.286	0.242 (0.154)	0.196 (0.471)	0.136	0.007 (0.009)	0.160 (0.294)	0.366
Statins	0.154 (0.322)	0.219 (0.394)	0.864	0.239 (0.491)	0.129 (0.184)	0.676	0.103 (0.134)	0.276 (0.479)	0.531
Warfarin	0.077 (0.119)	0.179 (0.351)	0.929	N/A	0.206 (0.422)	N/A	0.077 (0.119)	0.161 (0.299)	0.868

*Note. Area percentage of OM is presented as mean (±SD). Information of each clinical parameter was available for all the patients unless otherwise stated. Association of amount of OM in a slide to categorical clinical parameters calculated by Mann–Whitney U test*.

*“Yes” refers to the patients having the clinical characteristic, and “No” to the patients not having the clinical characteristic*.

*OM osteoid metaplasia; LEAD, lower extremity artery disease*.

a *Overall comparison by Kruskal–Wallis test between all the groups*.

b *“Yes” for claudication and “no” for rest pain/ulcer/gangrene*.

**Table 4:** Association of the area percentage of OM to continuous baseline characteristics.

**Characteristic**	**All patients**		**Female**		**Male**	
	**rho**	***p***	**rho**	***p***	**rho**	***p***
Age (years)	−0.003	0.979	−0.125	0.481	0.054	0.695
BMI (kg/m^2^)	0.109	0.355	0.129	0.506	0.092	0.546
Ankle brachial index (*n* = 76)	−0.072	0.534	0.160	0.417	−0.187	0.203
Toe pressure (mmHg) (*n* = 76)	−0.031	0.790	0.309	0.103	−0.296	0.044
Hemoglobin (g/L) (*n* = 88)	−0.017	0.872	−0.136	0.452	0.062	0.652
Total leukocyte count (10E9/L) (*n* = 88)	−0.006	0.954	−0.048	0.793	0.040	0.772
Thrombocytes (10E9/L) (*n* = 88)	−0.035	0.748	−0.191	0.286	0.048	0.727
Total cholesterol (mmol/L) (*n* = 88)	0.119	0.270	−0.044	0.809	0.220	0.104
Low-density lipoprotein-cholesterol (mmol/L) (*n* = 87)	0.096	0.374	0.002	0.994	0.150	0.270
High-density lipoprotein-cholesterol (mmol/L) (*n* = 88)	0.073	0.498	−0.029	0.876	0.062	0.649
Triglycerides (mmol/L) (*n* = 88)	0.002	0.988	−0.232	0.202	0.140	0.305
High-sensitivity C-reactive protein (mg/L) (*n* = 88)	0.038	0.725	−0.088	0.634	0.144	0.291
Glomerular filtration rate (μmol/L) (*n* = 88)	0.083	0.441	0.159	0.378	0.112	0.417

*Note. Information of each clinical parameter was available for all the patients unless otherwise stated. Association of the area percentage of OM in a slide to continuous clinical parameters calculated by Spearman's rank correlation test*.

*OM, osteoid metaplasia; BMI, body mass index; rho, Spearman's rank correlation coefficient*.

In the categorical analysis, males with most OM had more severe clinical symptoms (rest pain and ulcer) in comparison with the males without OM (OR = 4.857; 95%CI = 1.212–19.466, *p* =0.022). TP was inversely associated with a higher area percentage of OM (the highest group vs. two lower groups) in male patients (*p* = 0.022) (Supplementary Table II and III).

We performed a logistic regression analysis to analyze the association between male patients' symptoms and the area percentage of OM in a slide having symptoms (claudication and rest pain, ulcer or gangrene) as a dependent variable and the area percentage of OM (the lowest, middle, and highest groups) as an explanatory variable. The regression model included 46 males and was adjusted for TP and blood thrombocyte count. ABI, LDL-C, and hs-CRP were also significantly associated with symptoms (Table 5), but because they correlated strongly with TP (rho = 0.756, *p* < 0.0001 for ABI, rho = 0.347, *p* = 0.017 for LDL-C, rho = 0.409, *p* = 0.004 for hs-CRP), which had the strongest correlation to symptoms, they were not included in the logistic regression model. As a result, a higher area percentage of OM and lower TP were both independently associated with more severe clinical disease (rest pain and ulcer) in male patients (OR = 9.426; 95%CI = 1.168–76.037, *p* = 0.035 for OM and OR = 0.909; 95%CI = 0.852–0.970, *p* = 0.004 for TP) (Table 6).

**Table 5:** Associations between symptoms (claudication vs. rest pain or ulcer) and clinical parameters in male patients.

	**Claudication**	**Rest pain or Ulcer**	***P***	**OR**	**95%CI**
N	35	21			
Age (years)	68.41 [8.73]	68.76 [11.59]	0.886		
BMI (kg/m^2^) (*n* = 45)	26.95 [6.00]	28.26 [7.00]	0.516		
Previous invasive treatments for LEAD	16 (45.7)	8 (38.1)	0.577		
Ankle brachial index (*n* = 48)	0.50 [0.21]	0.30 [0.29]	<0.0001		
Toe pressure (mmHg) (*n* = 47)	52.00 [26.00]	34.50 [23.00]	<0.0001		
**Risk factors**					
Smoking status					
Never	0 (0.0)	0 (0.0)	–		
Current smoker	12 (35.3)	9 (42.9)	0.575		
Ex-smoker	22 (64.7)	12 (57.1)	0.583		
Diabetic	13 (37.1)	12 (57.1)	0.145		
Hypertensive	28 (80.0)	20 (95.2)	0.235		
Dyslipidemia	32 (91.4)	20 (100.0)	0.293		
Coronary artery disease	12 (34.3)	8 (38.1)	0.773		
Cerebrovascular disease	2 (5.7)	2 (9.5)	0.626		
**Medications**					
ACE inhibitors/ATR blockers	26 (74.3)	19 (90.5)	0.179		
Aspirin	27 (77.1)	13 (61.9)	0.222		
Clopidogrel	2 (5.7)	0 (0.0)	0.523		
Statins	25 (71.4)	15 (71.4)	1.000		
Warfarin	2 (5.7)	2 (14.3)	0.352		
**Laboratory results**					
Hemoglobin (g/L) (*n* = 55)	144.00 [17.00]	133.00 [32.50]	0.150		
Total leukocyte count (10E9/L) (*n* = 55)	7.40 [2.30]	8.30 [1.70]	0.101		
Thrombocytes (10E9/L) (*n* = 55)	222.50 [86.30]	281.00 [76.00]	0.017		
Total cholesterol (mmol/L)	3.70 [1.10]	4.30 [1.35]	0.058		
Low-density lipoprotein-cholesterol (mmol/L) (*n* = 56)	1.80 [0.80]	2.30 [1.20]	0.005		
High-density lipoprotein-cholesterol (mmol/L) (*n* = 56)	1.06 [0.40]	1.14 [0.50]	0.774		
Triglycerides (mmol/L)	11.23 [1.22]	1.50 [1.50]	0.187		
High-sensitivity C-reactive protein (mg/L)	0.99 [1.54]	4.47 [8.48]	<0.0001		
Glomerular filtration rate (μmol/L) (*n* = 55)	87.77 [18.77]	82.45 [33.54]	0.835		
Area percentage of OM (categorical)			0.023^a^		
Lowest group	17 (48.6)	5 (23.8)			
Middle group	11 (31.4)	6 (28.6)	0.030^b^	3.636	1.105–11.969
Highest group	7 (20.0)	10 (47.6)	0.022^c^	4.857	1.212–19.466

*Note. Data are presented as median [IQR] or number of study subjects (%). Information of each clinical parameter was available for all male patients unless otherwise stated*.

*OM, osteoid metaplasia; BMI, body mass index; LEAD, lower extremity artery disease*.

a *Linear-by-linear association*.

b *The middle group vs. the lowest group*.

c *The highest group vs. the lowest group*.

**Table 6:** Binary logistic regression analysis of the associations between symptoms and area percentage of OM in male patients.

**OM group**	***N***	**Area percentage of OM range**	***p*^a^**	**OR**	**95%CI**	***p*^b^**	**Adjusted OR**	**95%CI**	***p*^c^**	**Multivariable Adjusted OR**	**95%CI**
Lowest	18	0		Ref.			Ref.				
Middle	14	0.01–0.17	0.112	3.750	0.735–19.140	0.053	9.445	0.969–92.038	0.143	6.514	0.532–79.741
Highest	14	0.18–1.43	0.009	9.000	1.724–46.994	0.035	9.426	1.168–76.037	0.029	15.892	1.330–189.884

*OM, osteoid metaplasia; ref., reference category*.

a *p-value for unadjusted regression model*.

b *p-value for adjusted regression model (adjusted for toe pressure and thrombocytes)*.

c *p-value for multivariable adjusted regression model (adjusted for toe pressure, thrombocytes, age, diabetes, cigarette smoking, and hypertension)*.

The multivariable adjusted logistic regression model, adjusted for TP, thrombocytes, and cardiovascular risk factors (age, diabetes, dyslipidemia, cigarette smoking, and hypertension) showed that only TP was associated with the symptoms of LEAD (OR = 0.886; 95%CI = 0.804–0.976, *p* = 0.014). We also assessed the associations between OM and symptoms by multivariable adjusted logistic regression analysis without dyslipidemia. As a result, both OM and TP were associated with symptoms (OR = 15.892; 95%CI = 1.330–189.844, *p* = 0.029 for OM and OR = 0.895; 95%CI = 0.823–0.974, *p* = 0.010 for TP).

## Discussion

Our results show that OM is a common finding in femoral artery plaques. The use of a DL algorithm is suitable for the research of atherosclerotic plaque tissue. The algorithm finds OM areas in the plaque and provides precise data, i.e., an area percentage for each section. The area percentage of OM was significantly associated with the severity and clinical manifestation of LEAD, as depicted by the significant association with TP and symptoms, in male patients. In female patients, a significant association was observed with OM area and diabetes.

The use of digital microscopy is becoming increasingly frequent in the field of medicine, especially pathology, and it enables the use of machine learning algorithms in the analysis of tissue samples. Previously, DL-based AI algorithms have been successfully used to analyze samples of tumor and dermatological tissues (6, 26). The analysis conducted with the help of the DL algorithms can achieve similar levels of accuracy as those achieved through visual assessments performed by pathologists (6, 27). The use of a DL-based algorithm is a novel approach in the research of vascular disease; however, there is evidence of the advantages of using a quantitative image analysis tool in the study of atherosclerotic plaques (28). Recent research has focused on improving imaging technology to identify possible prognostic factors of atherosclerotic lesions (29). However, the study of the atherosclerotic plaque specimens using DL tools provides more detailed information of the histological structure and a possibility to identify disease patterns that may be overlooked by visual inspection (7).

According to our results, the DL algorithm is a suitable tool for detection of OM in the atherosclerotic plaque, although in this study visual confirmation was used to finalize the results. Since this method has not been applied to atherosclerotic tissue before, we decided to confirm the results of the algorithm by visual detection by two scientists. This revealed that the most problematic tissue structure for the algorithm was the lamellar structure of thrombus formed by mechanical shear during the sectioning of the samples that closely resembles the lamellar structure of OM. Artifacts caused by cutting of the samples have been described before as a challenge for WSI analysis (30). This problem can be overcome with more examples of the challenging tissue entities in the training set. The annotation of even more areas of lamellar-appearing thrombus as not containing OM will improve false-positive discrimination. Consequently, the need for visual confirmation of results will decrease. In this study, visual confirmation of the results allowed us to acknowledge the shortcomings of the algorithm. We decided to include the slides that clearly had OM in the analysis and discard those that were definitively false-positive results by visual confirmation in order to not skew the results of the analysis. Nevertheless, without the use of the algorithm, precise quantitative analysis of OM in the plaques would be impossible.

Several recent studies have addressed the issue of OM in patients with LEAD; however, using conventional methods, OM has not been related to the severity of the disease (2, 15, 31). Our results indicate that a higher area percentage of OM is associated with severity of clinical symptoms of LEAD in male patients. This association did not reach statistical significance when we compared males with OM to males without OM, but it emerged when we analyzed the area percentage of OM as a continuous variable or an ordinal variable based on the continuous one. This probably explains why OM in femoral artery plaques has not, to our knowledge, been linked to clinical symptoms in patients with LEAD before.

The greater amount of OM in the plaques of patients with severe symptoms may be explained by the further advancement of the disease in these patients. This may indicate that in LEAD the patients with most severe symptoms are those with advanced disease locally causing an occlusion of arterial blood flow. Thus, the role of plaque rupture in LEAD may be of lesser consequence compared with carotid artery disease where the most severe symptoms are caused by plaque rupture and OM in the plaque has been reported to be a stabilizing feature.

ABI and TP are noninvasive tests used in the diagnosis of LEAD that describe the overall atherosclerotic burden of the lower limb arteries (32). ABI and especially TP decrease in advanced LEAD as a sign of lowered perfusion of the affected limb (33). Accordingly, both were inversely associated with symptoms in our study.

In female patients, we did not find any association between OM and clinical symptoms, which might be due to the small sample size or the possible differences in clinical manifestations of LEAD between men and women. It has been reported that women with LEAD have greater functional impairment than men with the same ABI level (34). Gender differences in atherosclerotic disease progression and symptoms may be due to hormonal differences and specifically the role of estrogen in the female metabolism (35).

In female patients, the presence of OM was associated with diabetes. All the diabetic women had OM, corroborating the previously published data reporting a higher prevalence of calcified plaques and OM in a plaque in diabetic patients (16, 36). Vascular calcification resembles osteogenesis, when vascular smooth muscle cells (VSMCs) express bone-associated proteins (e.g., osteocalcin, osteopontin, bone sialoprotein, and alkaline phosphatase) that promote the calcification processes (37). Previously, it was shown that high glucose levels accelerate osteogenic processes (38). Furthermore, these bone-associated proteins have been detected in histologic sections of vessels in patients with diabetes and end-stage renal disease (39). Notably, diabetes was not associated with OM in male patients.

Our study has several limitations. The analysis included only a single 4-μm-thick segment of each plaque, and therefore, the sample might not represent all structures or their extent in the plaque. The sample is from the thickest and central and therefore the most advanced and stable portion of the plaque, since the composition of the edges of the plaque may be compromised due to erosion and ongoing inflammatory processes (32). Thus, an assessment of the advancement of the disease in terms of plaque features can be made from the segment.

The small sample size limited the validity of subgroup analyses. Clinical characteristics were collected from the electronic patient records and included therefore only those details concerning each patient that had been documented. Also, we have not included the atherosclerotic lesions in the aortoiliac or infrainguinal arteries into the analysis. The results of this study reflect findings in patients with severe LEAD, and the results cannot be generalized to concern all LEAD patients.

The exact quantification of OM using a DL-based algorithm reveals novel associations with clinical characteristics that have not been shown before using conventional microscopy. This provides interesting perspectives for the future, where the algorithm may be applied to a larger set of samples and samples from other vascular beds as well. Furthermore, analyzing a larger set of samples from a single patient will allow for a more thorough study of a single patient's plaque characteristics. Building an algorithm to characterize other features of the plaques may also provide new insight into the mechanisms of atherosclerotic plaque formation and progression and may advance the gender-specific understanding of the progression of atherosclerotic disease. In addition, the clinical significance of specific plaque characteristics in the treatment of atherosclerotic lesions warrants further research.

## Conclusions

Our study presents a novel approach to atherosclerotic plaque research by applying a DL algorithm. The use of the algorithm provided exact amounts of OM in the studied sections of femoral artery plaques. OM is a common finding in atherosclerotic plaques in the femoral artery. The area percentage of OM in the plaques was associated with diabetes in female patients and severity of LEAD in male patients. Our results suggest a gender difference in the association of OM with clinical severity of lower limb ischemia, revealed by the use of a DL-based image analysis tool, urging further research on the gender-specific etiological processes and clinical manifestations of LEAD.

## Data Availability Statement

The raw data supporting the conclusions of this article will be made available by the authors, without undue reservation.

## Ethics Statement

The study was reviewed and approved by The Ethics Committee of the Hospital district of Helsinki and Uusimaa, Helsinki, Finland. The patients/participants provided their written informed consent to participate in this study.

## Author Contributions

ML, JT, and AL analyzed the data and drafted the manuscript. ML and AL built the image analysis algorithm. ML and JT performed the statistical analyses. JT, AA, MV, MM, and JS collected the data. ML, JT, and RP gathered clinical characteristics of the patient cohort and NL and JL are specialists in deep learning algorithm analysis and provided technical assistance. M-LL and JS supervised the study project. All authors contributed to study design, revised the manuscript and approved the final manuscript.

## Conflict of Interest

JL is a founder and consultant at Aiforia Ltd. The remaining authors declare that the research was conducted in the absence of any commercial or financial relationships that could be construed as a potential conflict of interest.
