# Supplementary material for: Osteoid Metaplasia in Femoral Artery Plaques Is Associated With the Clinical Severity of Lower Extremity Artery Disease in Men
Source: Front Cardiovasc Med. 2020 Dec 10;7:594192. doi: 10.3389/fcvm.2020.594192 (PMC7758249; doi:10.3389/fcvm.2020.594192)
Supplement: Supplementary file 1 [file Data_Sheet_1.PDF]

Supplementary figures

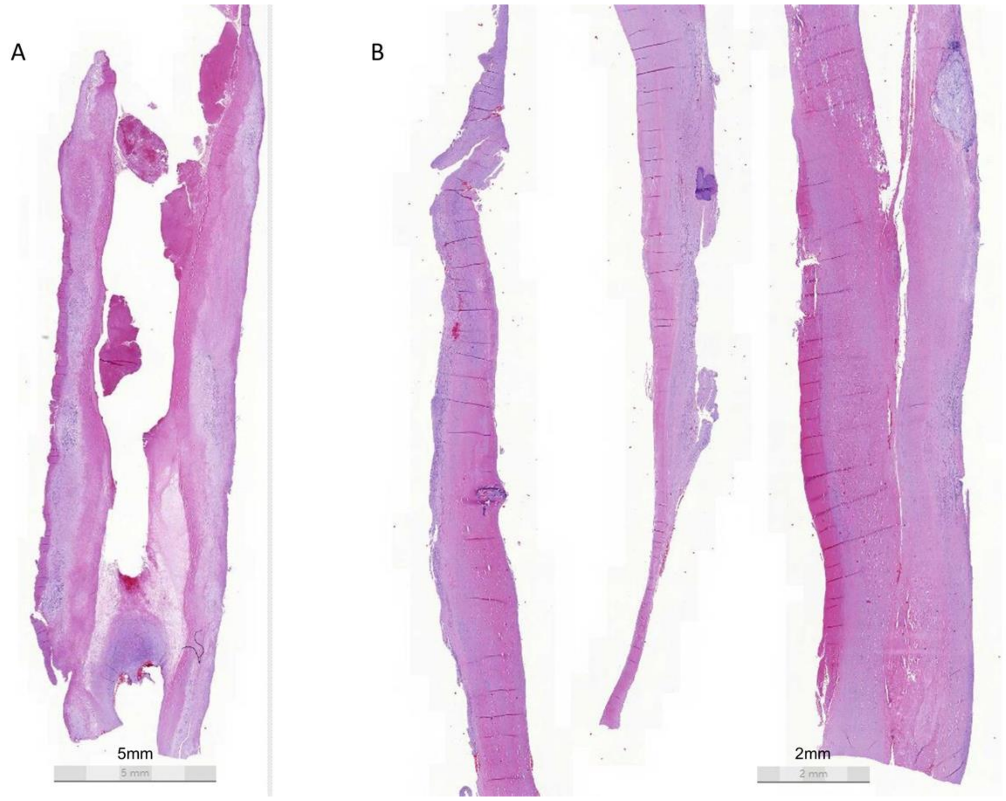

Supplementary figure I. H&E stained slides of plaques from 2 patients showing a low level of dysmorphia and no osteoid metaplasia (OM). (A) An overview of the whole slide. (B) A magnified view of a plaque with little dysmorphia.

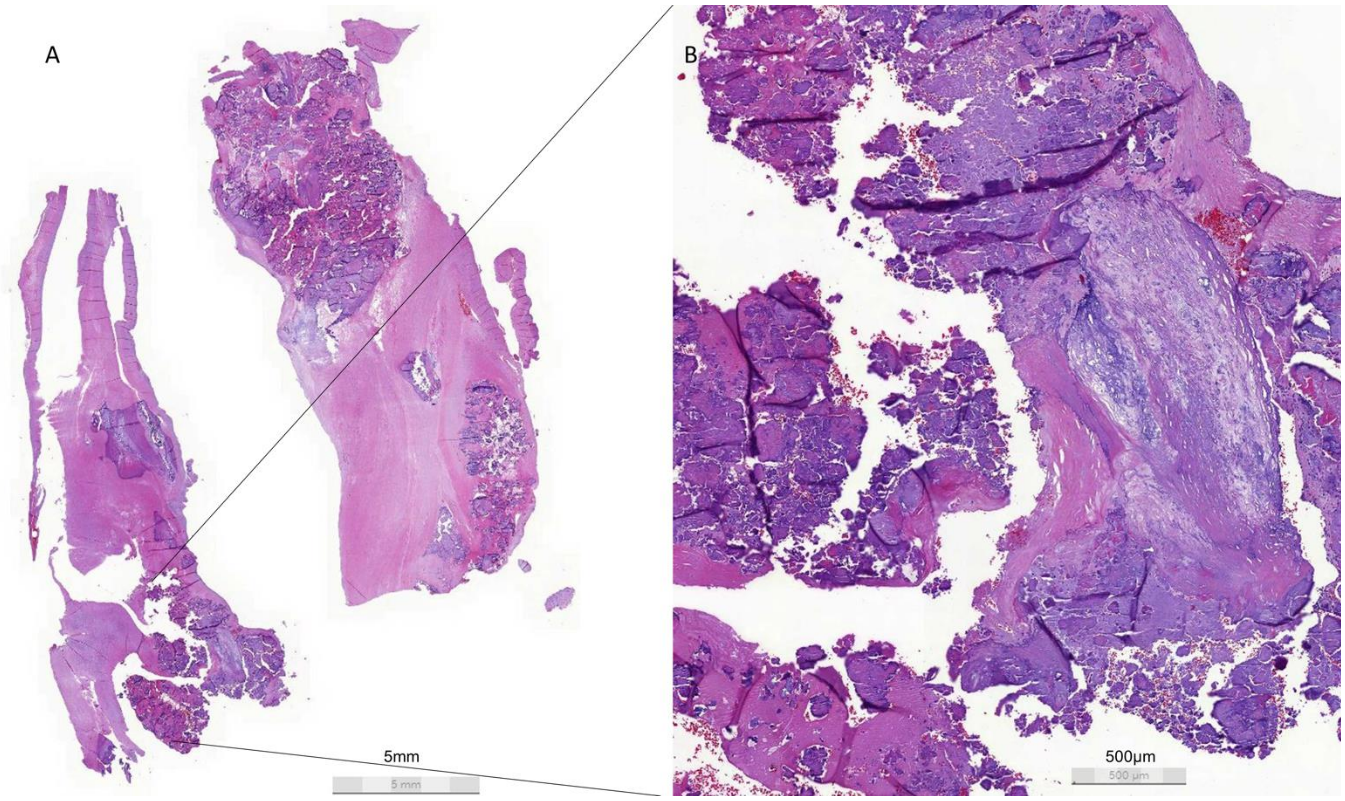

Supplementary figure II. An example of a plaque from one patient showing a high level of dysmorphia and calcification but no OM. (A) Overview of the slide. (B) Magnified view of a dysmorphic and calcified region.

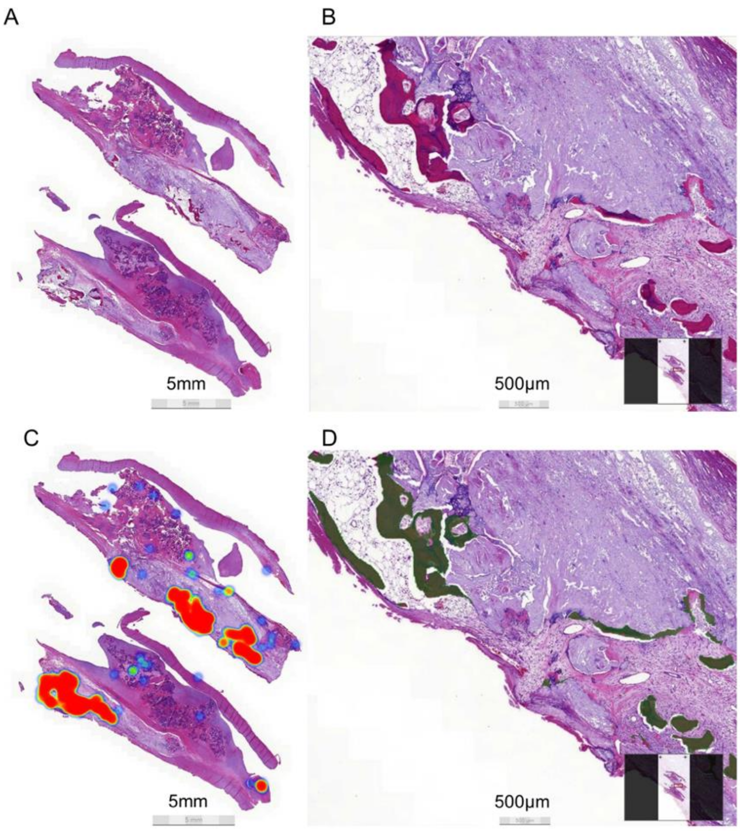

Supplementary figure III. A femoral artery plaque containing osteoid metaplasia (OM) from one patient. (A) Overview of the plaque structure revealing significant dysmorphia of the vessel wall. (B) A magnified view of the outer section of the slide showing osteoid metaplasia. (C) A heatmap view of the slide showing the areas in red that contain OM. (D) A magnified view of the same part of the slide as in B with OM areas in green as recognised by the algorithm.

Supplementary Table I

Differences in baseline characteristics between patients with OM and patients without OM among female and male patients

	Female					Male				
	OM-	OM+	*p*	OR	95%CI	OM-	OM+	*p*	OR	95%CI
n	17 (50.0)	17 (50.0)				22 (39.3)	34 (60.7)			
Age (years)	71.87 ± 7.02	70.84 ± 5.41	.617			66.57 ± 7.32	69.90 ± 6.99	.144		
BMI (kg/m2)	23.52 ± 15.39	24.66 ± 4.08	.380			27.61 ± 3.85	28.23 ± 18.50	.936		
Previous invasive treatments for LEAD	6 (35.3)	10 (58.8)	.169			13 (59.1)	11 (32.4)	.048	0.331	0.109-1.008
Severity of LEAD symptoms										
claudication	11 (64.7)	11 (64.7)	1.000			17 /77.3)	18 (52.9)	.066		
rest pain	4 (23.5)	4 (23.5)	1.000			3 (13.6)	7 (20.6)	.724		
ischemic ulcer or gangrene	2 (11.8)	2 (11.8)	1.000			2 (9.1)	9 (26.5)	.171		
Rest pain/ulcer/gangrene	6 (35.3)	6 (35.3)	1.000			5 (22.7)	16 (47.1)	.066		
Ankle brachial index	0.36 ± 0.10	0.41 ± 0.12	.285			0.46 ± 0.18	0.46 ± 0.24	.589		
Toe pressure (mmHg)	38.25 ± 24.20	46.92 ± 14.38	.043			48.47 ± 22.20	42.68 ± 18.24	.273		
Riskfactors										
Smoking status										
Never	13 (81.3)	16 (94.1)	.335			0 (0.0)	0 (0.0)	-		
Current smoker	8 (50.0)	10 (58.8)	.611			8 (36.4)	13 (39.4)	.821		
ex- smoker	5 (31.3)	6 (35.3)	.805			14 (63.6)	20 (60.6)	.821		
Diabetic	0 (0.0)	6 (35.3)	.018			10 (45.5)	15 (44.1)	.922		
Hypertensive	14 (82.4)	14 (82.4)	1.000			17 (77.3)	31 (91.2)	.241		
Dyslipidemia	14 (82.4)	15 (88.2)	1.000			20 (90.9)	32 (97.0)	.557		
Coronary artery disease	6 (35.3)	5 (29.4)	.714			6 (27.3)	14 (41.2)	.289		
Cerebrovascular disease	2 (11.8)	2 (11.8)	1.000			1 (4.5)	2 (8.8)	1.000		
Medications										
ACE inhibitors/ ATR blockers	10 (58.8)	12 (70.6)	.473			16 (72.7)	29 (85.3)	.310		
Aspirin	12 (70.6)	12 (76.5)	1.000			17 (77.3)	23 (67.6)	.436		
Clopidogrel	2 (11.8)	6 (35.3)	.225			1 (4.5)	1 (2.9)	1.000		
Statins	11 (64.7)	13 (76.5)	.452			16 (72.7)	24 (70.6)	.863		
Warfarin	0 (0.0)	0 (0.0)	-			1 (4.5)	4 (11.8)	.638		
Haemoglobin (g/l)	133.75 ± 11.98	130.65 ± 7.31	.407			140.76 ± 15.58	141.56 ± 19.04	.782		
Total leukocyte count (10E9/l)	8.11 ± 2.10	8.00 ± 1.50	.871			8.03 ± 1.94	7.81 ± 1.71	.945		
Thrombocytes (10E9/l)	289.44 ± 82.05	263.71 ± 52.99	.460			268.62 ± 88.80	258.56 ± 81.41	.883		
Total cholesterol (mmol/l)	4.91 ± 1.85	4.54 ± 0.96	.752			3.76 ± 0.84	4.04 ± 0.92	.239		
Low-density lipoprotein-cholesterol (mmol/l)	2.45 ± 0.69	2.53 ± 0.69	.830			1.98 ± 0.54	2.19 ± 0.83	.507		
High-density lipoprotein-cholesterol (mmol/l)	1.39 ± 0.35	1.48 ± 0.43	.956			1.13 ± 0.38	1.18 ± 0.39	.557		

Triglycerides (mmol/l)	2.21 ± 3.90	1.15 ± 0.24	.402		1.84 ± 1.79	1.74 ± 1.10	.580
High-sensitive C-reactive protein (mg/l)	2.10 ± 1.42	1.56 ± 1.14	.224		2.54 ± 3.46	5.65 ± 8.66	.149
Glomerular filtration rate (µmol/l)	71.12 ± 14.63	74.10 ± 15.00	.296		83.39 ± 15.92	80.53 ± 21.95	.986

Abbreviations: OM, osteoid metaplasia; BMI, body mass index; LEAD, lower extremity artery disease
OM-, patients without OM
OM+, patients with OM

Supplementary Table II

Differences in baseline characteristics between OM groups (lowest, middle, highest) in male patients

	Lowest	Middle	Highest	p_a	OR	95%CI	p_b	OR	95%CI	p_c
Area percentage of OM range (%)		0.01-0.17	0.18-1.43							
n=56	22 (39.3)	17 (30.4)	17 (30.4)							
Age (years)	66.57 ± 7.32	72.32 ± 5.10	67.48 ± 7.89	.977			.306			.667
BMI (kg/m2) (n=45)	27.61 ± 3.85	26.96 ± 3.40	29.71 ± 4.89	.320			.218			.571
Previous invasive treatments for LEAD	13 (59.1)	6 (35.3)	5 (29.4)	.065			.179			.059
Severity of LEAD symptoms				.067			.091			.030
claudication	17 (77.3)	11 (64.7)	7 (41.2)							
rest pain	3 (13.6)	2 (11.8)	5 (29.4)							
ischemic ulcer or gangrene	2 (9.1)	4 (23.5)	5 (29.4)							
Rest pain/ulcer	5 (22.7)	6 (35.3)	10 (58.8)	.022	4.86	1.212-19.466	.030	3.64	1.105-11.969	.023
Ankle brachial index (n=48)	0.46 ± 0.18	0.47 ± 0.21	0.45 ± 0.26	.409			.414			.473
Toe pressure (mmHg) (n=47)	48.47 ± 22.20	49.29 ± 19.21	36.07 ± 15.10	.043			.022			.067
Riskfactors										
Smoking status (n=55)				.501			.365			.531
Never	0 (0.0)	0 (0.0)	0 (0.0)							
Current smoker	8 (36.4)	5 (31.3)	8 (47.1)							
ex- smoker	14 (63.6)	11 (68.8)	9 (52.9)							
Diabetic	10 (45.5)	8 (47.1)	7 (41.2)	.789			.730			.805
Hypertensive	17 (77.3)	15 (88.2)	16 (94.1)	.206			.412			.134
Dyslipidemia (n=55)	20 (90.9)	16 (94.1)	16 (100.0)	.499			.548			.232
Coronary artery disease	6 (27.3)	7 (41.2)	7 (41.2)	.361			.573			.353
Cerebrovascular disease	1 (4.5)	1 (5.9)	2 (11.8)	.570			.577			.400
Medications										
ACE inhibitors/ ATR blockers	16 (72.7)	15 (88.2)	14 (82.4)	.704			1.000			.418
Aspirin	17 (77.3)	11 (64.7)	12 (70.6)	.721			1.000			.614
Clopidogrel	1 (4.5)	1 (5.9)	0 (0.0)	.590			1.000			.480
Statins	16 (72.7)	13 (76.5)	11 (64.7)	1.000			.527			.614
Warfarin	1 (4.5)	3 (17.6)	1 (5.9)	1.000			1.000			.803
Haemoglobin (g/l) (n=55)	140.76 ± 15.57	139.41 ± 2.26	143.71 ± 15.57	.681			.642			.670
Total leukocyte count (10E9/l) (n=55)	8.03 ± 1.94	7.89 ± 1.70	7.72 ± 1.76	.803			.927			.994
Thrombocytes (10E9/l) (n=55)	268.62 ± 88.80	260.65 ± 86.55	256.47 ± 78.55	.769			.709			.975
Total cholesterol (mmol/l) (n=56)	3.76 ± 0.84	3.97 ± 1.12	4.12 ± 0.69	.098			.127			.145

Low-density lipoprotein-cholesterol (mmol/l) (n=56)	1.98 ± 0.54	2.18 ± 1.06	2.19 ± 0.54	.256	.296	.381
High-density lipoprotein-cholesterol (mmol/l) (n=56)	1.13 ± 0.38	1.20 ± 0.36	1.17 ± 0.43	.944	.859	.734
Triglycerides (mmol/l) (n=56)	1.84 ± 1.79	1.55 ± 0.65	1.94 ± 1.41	.453	.417	.433
High-sensitive C-reactive protein (mg/l) (n=56)	2.54 ± 3.46	5.45 ± 8.99	5.85 ± 8.59	.223	.349	.150
Glomerular filtration rate (µmol/l) (n=55)	83.39 ± 15.92	73.38 ± 23.50	87.68 ± 18.25	.352	.122	.425

[a] highest vs lowest group

[b] highest vs middle + lowest groups

[c] Linear-by-Linear association for categorical variables and Jonckheere-Terpstra test for trend for continuous variables

Abbreviations: OM, osteoid metaplasia; BMI, body mass index; LEAD, lower extremity artery disease

Supplementary Table III

Differences in baseline characteristics between OM groups (lowest, middle, highest) in female patients

	Lowest	Middle	Highest	p_a	OR	95%CI	p_b	OR	95%CI	p_c
Area percentage of OM range (%)		0.05-0.23	0.24-2.32							
n=34	17 (50.0)	8 (23.5)	9 (26.5)							
Age (years)	71.87 ± 7.02	70.82 ± 7.01	70.86 ± 3.94	.634			.788			.685
BMI (kg/m2) (n=29)	23.52 ± 3.92	25.54 ± 3.55	23.26 ± 4.90	.842			.594			.585
Previous invasive treatments for LEAD	6 (35.3)	3 (37.5)	7 (77.8)	.097			.052			.055
Severity of LEAD symptoms				NS			NS			.946
claudication	11 (64.7)	5 (62.5)	6 (66.7)							
rest pain	4 (23.5)	2 (25.0)	2 (22.2)							
ischemic ulcer or gangrene	2 (11.8)	1 (12.5)	1 (11.1)							
Rest pain/ulcer	6 (35.3)	3 (37.5)	3 (33.3)	1.000			1.000			.941
Ankle brachial index (n=28)	0.36 ± 0.10	0.46 ± 0.12	0.38 ± 0.12	.769			.959			.447
Toe pressure (mmHg) (n=29)	38.25 ± 24.20	52.40 ± 14.88	43.50 ± 13.90	.172			.487			.109
Riskfactors										
Smoking status (n=33)				NS			NS			.430
Never	3 (18.8)	1 (12.5)	0 (0.0)							
Current smoker	8 (50.0)	4 (50.0)	6 (66.7)							
ex- smoker	5 (31.3)	3 (37.5)	3 (33.3)							
Diabetic	0 (0.0)	4 (50.0)	2 (22.2)	.111			.644			.073
Hypertensive	14 (82.4)	7 (87.5)	7 (77.8)	1.000			.644			.828
Dyslipidemia (n=)	14 (82.4)	8 (100.0)	7 (77.8)	1.000			.591			.920
Coronary artery disease	6 (35.3)	4 (50.0)	1 (11.1)	.357			.214			.301
Cerebrovascular disease	2 (11.8)	1 (12.5)	1 (11.1)	1.000			1.000			.971
Medications										
ACE inhibitors/ ATR blockers	10 (58.8)	6 (75.0)	6 (66.7)	1.000			1.000			.621
Aspirin	12 (70.6)	7 (87.5)	6 (66.7)	1.000			.670			.957
Clopidogrel	2 (11.8)	2 (25.0)	4 (44.4)	1.000			.165			.066
Statins	11 (64.7)	7 (87.5)	6 (66.7)	.138			1.000			.776
Warfarin	0 (0.0)	0 (0.0)	0 (0.0)	-			-			-
Haemoglobin (g/l) (n=33)	133.75 ± 11.98	131.13 ± 5.19	130.22 ± 9.12	.452			.486			.370
Total leukocyte count (10E9/l) (n=33)	8.11 ± 2.10	8.01 ± 1.35	7.92 ± 1.71	.846			.736			.748
Thrombocytes (10E9/l) (n=33)	289.44 ± 82.05	266.25 ± 53.36	261.44 ± 55.79	.419			.538			.685
Total cholesterol (mmol/l) (n=32)	4.19 ± 1.85	4.47 ± 1.04	4.59 ± 0.95	1.000			.869			.873
Low-density lipoprotein-cholesterol (mmol/l) (n=31)	2.45 ± 0.69	2.37 ± 1.14	2.66 ± 0.92	.770			.593			.941

High-density lipoprotein-cholesterol (mmol/l) (n=32)	1.39 ± 0.35	1.51 ± 0.55	1.46 ± 0.35	.934	.934	.943
Triglycerides (mmol/l) (n=32)	2.21 ± 3.90	1.26 ± 0.22	1.07 ± 0.23	.207	.122	.183
High-sensitive C-reactive protein (mg/l) (n=32)	2.10 ± 1.42	1.25 ± 0.75	1.80 ± 1.37	.598	.902	.347
Glomerular filtration rate (µmol/l) (n=33)	71.12 ± 14.63	74.03 ± 16.76	74.16 ± 14.29	.388	.564	.345

[a] highest vs lowest group

[b] highest vs middle + lowest groups

[c] Linear-by-Linear association for categorical variables and Jonckheere-Terpstra test for trend for continous variables

Abbreviations: OM, osteoid metaplasia; BMI, body mass index; LEAD, lower extremity artery disease
